# Clavipectoral Fascia Plane Block Combined With Superficial Cervical Plexus Block for the Removal of Osteosynthesis Material From Clavicle Fracture

**DOI:** 10.7759/cureus.43146

**Published:** 2023-08-08

**Authors:** Delilah Gonçalves, Cristina P Sousa, Rita Graça, Maria P Miguelez, Catarina Sampaio

**Affiliations:** 1 Anesthesiology Department, Centro Hospitalar Trás-os-Montes e Alto Douro, Vila Real, PRT

**Keywords:** supraclavicular nerve, superficial cervical plexus, clavicle surgery, regional anesthesia, clavipectoral fascia block

## Abstract

Clavipectoral fascial plane block combined with superficial cervical plexus block has been used as an anesthetic and analgesic technique in mid-clavicle fracture surgeries. The authors describe two cases in which patients underwent extraction of osteosynthesis material from the clavicle, using clavipectoral fascial plane block combined with superficial cervical plexus block as an anesthetic and analgesic technique in the postoperative period. The mentioned block presented itself as an easy-to-perform technique, apparently safe and effective, allowing to obtain satisfactory results.

## Introduction

The clavipectoral fascia plane block was described for the first time at the 36th Congress of the “European Society of Regional Anesthesia and Pain Therapy,” in 2017, by Valdez [1-4]. In 2019, during the 44th Annual Meeting of the “Regional Anesthesiology and Acute Pain Medicine,” Roqués described the clavipectoral fascia plane block combined with the superficial cervical plexus block, as an anesthetic technique, highlighting its potential utility, in addition to post-operative analgesia of fractures of the middle third of the clavicle [1].

The innervation of the clavicle has been described as complex and controversial since there is great individual variability [2,4-6] The subscapular, axillary, suprascapular, accessory spinal, subclavian, and lateral pectoral nerves are responsible for the sensory innervation of the clavicle periosteum [2,5-8]. These reach the clavicle, penetrating the clavipectoral fascia [1,5]. As for the sensory innervation of the skin that covers the clavicle, the upper portion of the chest, and the shoulder, it is the responsibility of the supraclavicular nerve of the superficial cervical plexus [2,6].

The authors describe two cases in which clavipectoral fascia plane block was performed, complemented with superficial cervical plexus block, as an anesthetic and analgesic technique in the postoperative period, both in patients undergoing extraction of osteosynthesis material from the clavicle.

 One of the cases was previously presented as poster at the ESRA Virtual Congress on September 8-10, 2021.

## Case presentation

**Figure 1:**
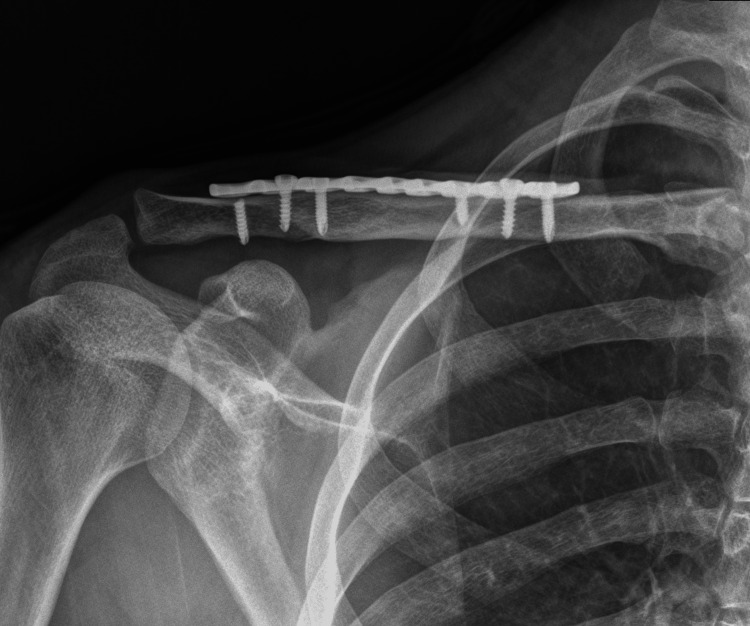
X-ray of the clavicle with the osteosynthesis material.

Two patients, previously submitted to corrective surgery for clavicle fracture, under balanced general anesthesia, were readmitted for extraction of osteosynthesis material (Figure 1).

In both cases, informed consent was obtained to perform the procedure under regional anesthesia, proceeding to block the clavipectoral fascia plane, complemented with the blockade of the supraclavicular nerve of the superficial cervical plexus. After positioning the patients in the supine position, with the head oriented to the contralateral side to the block, the ultrasound-guided technique was performed under aseptic conditions, with the Vivid I portable ultrasound (GE Healthcare, Germany), using a high frequency linear probe (12 MHz) and Echoplex+ (Vygon®, France) needle of 22 G of 50 mm in length.

To perform the superficial cervical plexus block, we positioned the ultrasound probe transversely on the patient's neck, precisely at the midpoint of the sternocleidomastoid muscle. The needle was carefully introduced “in-plane,” moving from the lateral to medial direction, beneath the posterior border of the muscle and through the investing fascia, where the local anesthetic was then injected.

**Figure 2:**
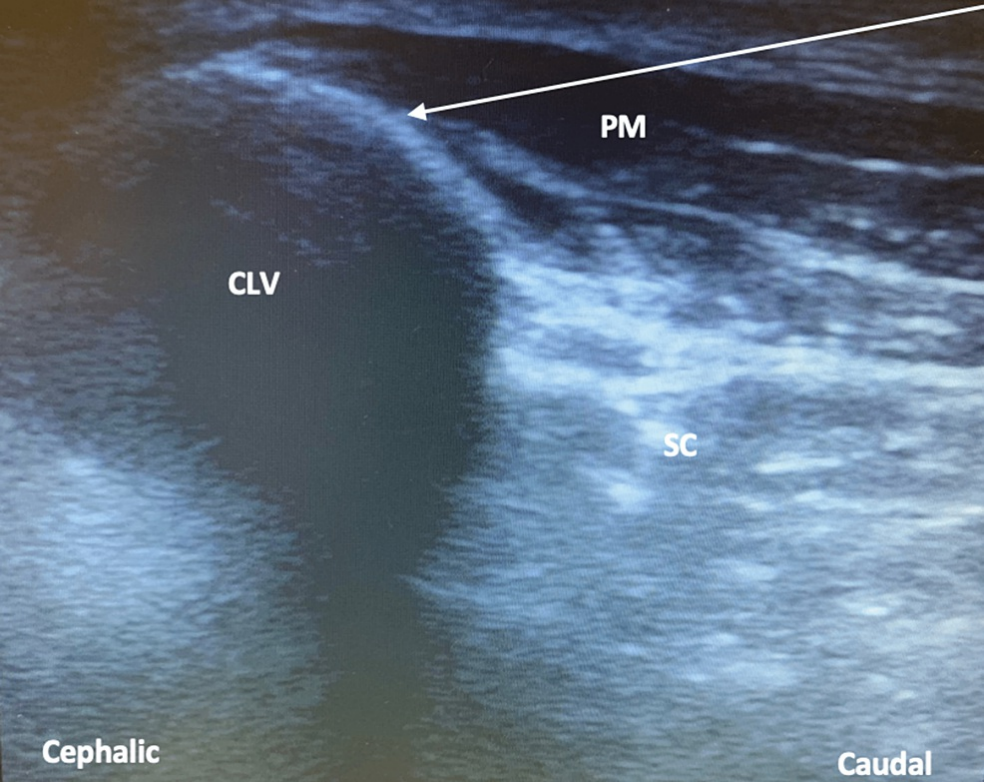
Ultrasonography image with representation of the needle and approach to the clavipectoral fascia plane block. CLV: Clavicle, PM: Pectoralis major muscle, SC: Subclavian muscle.

After, the clavipectoral fascia plane was blocked by administering local anesthetic, medial and lateral to the location of the osteosynthesis material. The ultrasound probe was placed perpendicular to the anterior surface of the clavicle; the needle was inserted “in-plane,” in a caudal to cephalic direction, in order to reduce the risk of pneumothorax. After ruling out the possibility of intravascular puncture and confirming the location of the needle with hydrodissection, the local anesthetic was administered between the periosteum of the clavicle and the clavipectoral fascia (Figure 2).

The first case refers to a male patient, 25 years old and weighing 70 kg, ASA I (physical health status of the American Society of Anesthesiologists), who showed a preference for regional anesthesia. Sedoanalgesia was instituted with an infusion of dexmedetomidine with a loading dose of 1 mcg.kg, for fifteen minutes followed by a maintenance dose of 0.5 mcg.kg.h. Intravenous 1 mg of midazolam and 50 mcg of fentanyl were also administered prior to the blockade.

For the blockade, 25 mL of local anesthetic (12.5 mL of 0.75% ropivacaine and 12.5 mL of 2% mepivacaine) were used, with 5 mL administered to block the superficial cervical plexus and the remaining 20 mL were administered between the periosteum of the clavicle and the clavipectoral fascia (10 mL laterally and the remaining 10 mL medially to the osteosynthesis material). During the blockade, 4 mg of dexamethasone were administered intravenously, in order to prolong the duration of the anesthetic effect.

The surgery lasted 30 minutes, during which 1 g of paracetamol, 100 mg of tramadol, 10 mg of metoclopramide and 30 mg of keterolac were administered as multimodal analgesia. During the entire surgical procedure, the patient remained pain-free and hemodynamically stable, with no complications recorded. After discharge from the post-anesthesia care unit, the patient was treated with the following postoperative analgesia: 1 g of intravenous paracetamol (6/6 hours) and 2 mg of morphine (4/4 hours), as rescue analgesia.

In the first 24 postoperative hours, the patient did not report pain, either at rest or with movement, not requiring rescue analgesia, until hospital discharge. The second case refers to a male patient, 47 years old and weighing 85 kg, ASA III, with a history of dyslipidemia and Brugada syndrome (with an implantable cardioverter-defibrillator). Before starting any anesthetic or surgical procedure, the cardioverter-defibrillator generator was turned off and the external defibrillator pads applied.

Prior to the blockade, sedoanalgesia was performed with 1 mg of midazolam and 100 mcg of intravenous fentanyl. Blockade was performed with 24 mL of 1% lidocaine and 4 mg of dexamethasone (1 mL). Of this solution, 5 mL was used to block the superficial cervical plexus and the remaining 20 mL was administered evenly on both sides of the osteosynthesis material, below the clavipectoral fascia.

During the surgery, lasting 25 minutes, 1 g of paracetamol and 30 mg of keterolac were administered. The patient did not report pain and no adverse events were recorded. The postoperative multimodal analgesia regimen included 1g of paracetamol (6/6 hours), 30 mg of keterolac (8/8 hours) and 2 mg of morphine (4/4 hours) for rescue analgesia.

In the first 24 postoperative hours, the patient did not complain of pain at rest or with movement. At 48 hours postoperatively, he presented mild pain with movement (VAS 2/10), however, without the need for rescue analgesia.

## Discussion

Despite its complexity and the numerous descriptions found in the literature, the clavipectoral fascia has been defined as a layer of connective tissue that surrounds the clavicle and the subclavian and pectoralis minor muscles. At a superior level, it extends laterally towards the coracoid process, at a medial level, it inserts into the anterior surface of the first to fifth ribs and, at an inferolateral level, it extends to the base of the axilla, forming part of the suspensory ligament of the axilla [1,9,10]. The clavipectoral fascia is perforated by the cephalic vein, thoracoacromial artery and vein, lateral pectoral nerve, and lymphatic vessels [9].

To date, the most commonly used anesthetic techniques for clavicle surgery are general anesthesia, alone or combined with peripheral nerve blocks. When regional anesthesia is the technique of choice, superficial cervical plexus block (combined or not with deep cervical plexus block) or interscalene or supraclavicular brachial plexus block is usually used [11].

For surgery to extract osteosynthesis material from the clavicle, the authors favor regional anesthesia, thus avoiding complications such as postoperative nausea and vomiting associated with general anesthesia, as well as the approach and difficulty of accessing the airway during the intraoperative. The patient's intolerance to the “Beach-chair” positioning could be considered a limitation to performing the surgery under regional anesthesia. However, as it was a short-term surgery, it was found that the discomfort was not significant.

The main complications associated with interscalene brachial plexus block are phrenic nerve block which can lead to respiratory function decline, particularly in patients with pulmonary conditions; Horner syndrome characterized by ptosis, myosis, and anhydrosis; and recurrent laryngeal nerve block resulting in dysphonia. The main complications inherent to supraclavicular brachial plexus block are also phrenic nerve block, Horner's syndrome (although less frequently than in interscalene block), and pneumothorax [8,9,11]. The authors chose to perform a clavipectoral fascial plane block, combined with a superficial cervical plexus block, to prevent the aforementioned complications.

The surgeries were performed successfully, with no evidence of complications. Furthermore, the patients remained hemodynamically stable, without pain (both intraoperatively and postoperatively) and without upper limb motor block. This last aspect contributed to patient satisfaction. Due to the success obtained, the authors consider that this approach could be an alternative to interscalene brachial plexus block for this type of intervention.

In the patient with Brugada syndrome, the authors considered regional anesthesia in order to avoid the sympathetic response associated with laryngoscopy, surgical incision, and extubation, preventing the risk of potentially fatal arrhythmias. During the procedure, no potentially harmful drugs were administered. The drug chosen for the blockade was a 1% lidocaine solution associated with dexamethasone. In total, 240 mg of lidocaine was administered, a dose lower than the toxic dose, for a patient weighing 85 kg, thus reducing the risk of systemic intoxication by local anesthetic and the consequent risk of arrhythmias. The surgery was uneventful, with no electrocardiographic changes. It should be noted that all materials and drugs considered safe, and necessary for conversion to general anesthesia, were properly prepared in case of block failure.

## Conclusions

In conclusion, the combination of the clavipectoral fascia block with the superficial cervical plexus block was presented as an easy-to-perform technique, apparently safe and effective. However, given the small number of cases described in the literature regarding the use of this block as an anesthetic technique in this type of surgery, the authors consider it a priority to carry out further research studies to test the efficacy and safety of this approach in patients proposed for clavicle surgery.
